# Dandelions, tulips and orchids: evidence for the existence of low-sensitive, medium-sensitive and high-sensitive individuals

**DOI:** 10.1038/s41398-017-0090-6

**Published:** 2018-01-22

**Authors:** Francesca Lionetti, Arthur Aron, Elaine N. Aron, G. Leonard Burns, Jadzia Jagiellowicz, Michael Pluess

**Affiliations:** 1 Queen Mary University of London, Mile End Rd, London, E14NS UK; 2 Stony Brook University, Stony Brook, NY 11794 USA; 3 Washington State University, Pullman, WA USA

## Abstract

According to empirical studies and recent theories, people differ substantially in their reactivity or sensitivity to environmental influences with some being generally more affected than others. More sensitive individuals have been described as orchids and less-sensitive ones as dandelions. Applying a data-driven approach, we explored the existence of sensitivity groups in a sample of 906 adults who completed the highly sensitive person (HSP) scale. According to factor analyses, the HSP scale reflects a bifactor model with a general sensitivity factor. In contrast to prevailing theories, latent class analyses consistently suggested the existence of three rather than two groups. While we were able to identify a highly sensitive (orchids, 31%) and a low-sensitive group (dandelions, 29%), we also detected a third group (40%) characterised by medium sensitivity, which we refer to as tulips in keeping with the flower metaphor. Preliminary cut-off scores for all three groups are provided. In order to characterise the different sensitivity groups, we investigated group differences regarding the Big Five personality traits, as well as experimentally assessed emotional reactivity in an additional independent sample. According to these follow-up analyses, the three groups differed in neuroticism, extraversion and emotional reactivity to positive mood induction with orchids scoring significantly higher in neuroticism and emotional reactivity and lower in extraversion than the other two groups (dandelions also differed significantly from tulips). Findings suggest that environmental sensitivity is a continuous and normally distributed trait but that people fall into three distinct sensitive groups along a sensitivity continuum.

Individual differences in reactivity or responsiveness to environmental stimuli have been observed across many species, including humans^1–4^. Such behavioural differences may be the function of inter-individual variability in the capacity for environmental sensitivity, recently defined as 'the ability to perceive and process environmental stimuli'^5^. Although this fundamental ability is relevant for all, given the importance of adaptation to specific environmental conditions for successful development, some individuals appear to be significantly more sensitive than others^6–8^. A vast number of studies have been chronicling such reactivity differences in over 100 species with a growing body of evidence for similar differences in humans emerging across the fields of psychology and psychiatry. For example, temperament traits (e.g. negative emotionality)^9^ as well as gene variants (e.g. short allele of the serotonin transporter gene polymorphism)^10^ have been associated with heightened sensitivity to the environment. Traditionally, these findings have been interpreted from a perspective of vulnerability informed by the diathesis stress model^11,12^. Central to this framework is the understanding that more reactive—or sensitive—individuals are more vulnerable to the negative effects of contextual adversity (e.g. childhood maltreatment, negative life events), while less reactive individuals prove to be resilient in the face of the same negative experience. However, this view has been challenged over the last decade by evolutionary-inspired theories according to which more reactive individuals may not only be more sensitive to the negative effects of adverse experiences, but also more sensitive to the beneficial effects of positive environmental exposures^1,5,13^. In other words, people may differ in their general sensitivity to both negative and positive environmental influences rather than exclusively in vulnerability to adverse experiences. Consequently, the leading theoretical frameworks on environmental sensitivity^7,14,15^ propose that higher sensitivity would be associated not only with increased vulnerability to adverse exposures but also with a heightened propensity to benefit from positive environmental influences, such as psychological intervention^16^. These theories further suggest that the majority of the general population would be characterised by lower and a minority by higher sensitivity^1^. These two distinctive patterns have been described in the popular orchid–dandelion metaphor^15^ according to which orchids represent those individuals who are generally more sensitive (i.e. they do exceptionally well in ideal conditions and exceptionally badly in poor ones) and dandelions, those who are generally less sensitive to environmental quality (i.e. they are resilient and can grow anywhere). In a recent paper, we reported a first investigation into the existence of sensitivity groups in children and adolescents^17^. Surprisingly, results across multiple samples suggested consistently that there were three rather than two groups. However, whether these distinct sensitivity prototypes also exist in adults has not been explored yet.

In this paper, we investigate the existence of different sensitivity groups in a large sample of 901 healthy adults based on their specific response patterns to the highly sensitive person (HSP) Scale^14^, an established self-report measure of environmental sensitivity. After testing with a confirmatory factor analysis whether the structure of the applied self-report measure reflects the hypothesised unidimensional general sensitivity trait, we examine whether the data support the existence of distinct sensitivity groups applying latent class analysis. We then propose preliminary cut-off scores that can be used to categorise individuals into the different detected sensitivity groups before exploring the profiles of the identified sensitivity groups in terms of differences in common personality traits (Big Five) and experimentally assessed emotional reactivity in an independent sample.

## Theories of environmental sensitivity

There are several theoretical frameworks for individual differences in sensitivity that emerged since the mid to late 1990s, with the most prominent being sensory processing sensitivity^6,14^, differential susceptibility theory^7,18,–21^ and biological sensitivity to context^13,15^. Each of these three concepts introduced unique theoretical insights regarding environmental sensitivity—discussed in more detail elsewhere^5,6,8^—while at the same time agreeing on two central aspects: (1) that sensitive individuals differ in their response to both negative and positive environmental influences, and (2) that a minority of the population is significantly more sensitive than the majority. While the first point is supported by a large number of empirical studies^7,21^ the second point is primarily based on theory^13,19^. However, according to the results of an innovative computer simulation study^1^, reactivity to the environment may be indeed higher in a minority due to its proposed negative frequency dependence (i.e. heightened sensitivity is only an advantage if not possessed by the majority). In line with these findings, a range of empirical studies suggest that traits associated with heightened sensitivity to environmental influences (e.g. difficult temperament, behaviour inhibition, sensitivity gene variants, etc.) tend to have a relatively low population frequency of about 10–35%^7,14,21–25^. In other words, there seems to be suggestive evidence that a minority of the population is characterised by high sensitivity, whereas the majority appears to be less sensitive (see also recent findings on the existence of sensitivity groups in children and adolescents^17^).

## Assessment of environmental sensitivity

Various markers of environmental sensitivity have been identified at different levels of analysis, including genetic, physiological and psychological factors^7^. However, these factors reflect more or less distal markers of sensitivity rather than representing a focused assessment of sensitivity. The only self-report measure specifically developed to assess general sensitivity in adults, as far as we know, is the HSP scale^14^. A growing number of studies provide evidence that the HSP scale is indeed measuring differences in environmental sensitivity. For example, behavioural studies report that individuals scoring high on the HSP scale are more responsive to both negative and positive stimuli^26–28^ and high HSP scores have been associated with greater activation in brain areas involved in higher-order visual processing in three separate studies^24,28,29^.

Originally, the HSP scale was designed to assess a one-dimensional construct of sensitivity—sensory processing sensitivity^14^. Several studies, though, found that data were more consistent with the existence of two, three or four factors^30–32^. The most consistent result is a three-factor solution with the following subscales: (a) ease of excitation (EOE), that is, being easily overwhelmed by external and internal stimuli (e.g. negative response to 'having a lot going at once' or performing worse at a task if observed); (b) aesthetic sensitivity (AES), capturing aesthetic awareness (e.g. being deeply moved by arts and music) and (c) low sensory threshold (LST), reflecting unpleasant sensory arousal to external stimuli (e.g. reaction to bright lights and loud noises). However, the typically moderate but significant correlations between the three HSP factors suggest that there may indeed exist a general trait of environmental sensitivity in addition to the three subscales. Hence, before exploring distinct sensitivity groups based on all items of the HSP scale, it is necessary to clarify whether there is any evidence for the existence of a general sensitivity factor.

## Overview of the current study

The main objectives of the current study are to investigate (a) whether environmental sensitivity as measured with the HSP Scale is indeed a unitary concept, testing a bifactor model within a confirmatory factor analysis framework; (b) whether HSP data support the existence of distinct sensitivity categories in the general population, applying a data-driven latent class analysis approach and identifying cut-off scores for the different detected sensitivity categories and finally (c), whether the detected sensitivity groups differ significantly regarding common personality traits and emotional reactivity.

## Methods

### Participants and procedure

The current study involves two samples, one for the primary objective of identifying sensitivity groups and a second independent sample for follow-up analyses in order to characterise the profile of the detected groups. The first sample included 906 psychology undergraduates at Stony Brook University (USA) who completed the HSP scale either as part of a standard 'mass testing' or during a lab visit. Ethical approval was obtained from Stony Brook University. Participants’ mean age was 19.20 (SD = 2.52) and 62.3% were female. The sample had the following ethnic distribution (based on data on ethnicity available for 66% of the sample): 44.6% Caucasian (non-Hispanic), 35.3% Asian American, 7.04% African American, 5.19% Hispanic and 7.9% mixed. Data from five participants had to be excluded due to incomplete questionnaires. For the remaining sample, the percentage of missing responses was computed for each item. Frequencies of any missing HSP items were <2%. The arithmetic mean of each item was used to replace missing data^33^. The sample was then randomly split in two subsamples (subsample A, *n* = 451 and subsample B, *n* = 450) to permit a cross-validation approach for testing and then re-testing whether the internal structure of the HSP scale can be considered unidimensional and to validate the cut-off scores.

The second sample featured data from 230 psychology undergraduates at Queen Mary University of London (UK). Participants were asked to complete a brief version of the HSP scale and a self-report measure of the Big Five personality traits. All 230 participants also took part in an experimental mood induction task in which they rated their mood before and after viewing a happy and a sad video clip. The two video clips were presented to all participants in randomised order to account for carry-over effects. Ethical approval was obtained from Queen Mary University of London. Participants’ mean age was 22.29 (SD = 5.47) and 69% were female. The sample had the following ethnic distribution: 42.61% Caucasian, 33.48% Asian, 6.96% African and 12.61% mixed/other.

### Measures

#### Sensitivity

Environmental sensitivity was assessed in the primary sample with the 27-item HSP scale. Each item was rated on a 7-point Likert scale ranging from '1 = strongly disagree', to '7 = strongly agree'. Example items are 'Are you easily overwhelmed by strong sensory input?', 'Do other people’s mood affect you?', 'Are you deeply moved by arts or music?'. The mean score across all items was computed in order to create the total score with higher scores reflecting higher sensitivity. Cronbach’s alpha was high with *α* = 0.89 [95% CI 0.88–0.90]. In the follow-up sample, environmental sensitivity was measured with a brief 12-item version of the HSP scale, which is characterised by comparable psychometric and construct validity properties^34^.

#### Personality

The Big Five personality traits in the follow-up sample were assessed with 50 items from the international personality item pool^35^. Each of the five personality traits was assessed with 10 statements (e.g. for extraversion: 'I feel comfortable around people', for neuroticism: 'I get stressed out easily'), rated on a five-point scale ranging from 1 = 'very inaccurate' to 5 = 'very accurate'. Internal consistency was good with *α* = 0.90 [95% CI 0.88–0.92] for extraversion, *α* = 0.81 [95% CI 0.80–0.83] for agreeableness, *α* = 0.79 [95% CI 0.75–0.83] for conscientiousness, α = 0.84 [95% CI 0.82–0.88] for neuroticism and *α* = 0.78 [95% CI 0.73–0.82] for openness.

#### Emotional reactivity

In the follow-up sample, negative and positive emotional reactivity scores were created with the help of an experimental mood induction task involving sad and happy video clips. Participants rated their mood before and after each video clip on a visual analogue scale ranging from 0 = 'Not happy at all/Not sad at all' to 100 = 'Very Happy/Very Sad'. Pre-video scores were then subtracted from post-video scores in order to compute a negative emotional reactivity score related to the sad video and a positive emotional reactivity score in relation to the happy video clip.

### Data analysis

#### Preliminary analysis

In the primary sample, we first explored the distribution of the HSP mean score with a density plot. Then, we compared alternative HSP factorial models with a series of confirmatory factor analyses (CFA): one-factor, three-factor and bifactor. Each model was tested in subsample A and then retested in subsample B. We did not test the fit of a second-order model (three dimensions plus a common higher-order factor) because this model would have produced identical fit to the correlated three-factor model^36^. In the one-factor model, all items were allowed to load on one general sensitivity factor; in the three-factor model (derived from ref. 32), each item loaded only on one of three specific factors and factors were allowed to correlate; in the bifactor model, factors were constrained to be orthogonal (i.e. uncorrelated), and each item was allowed to load both on a specific factor and on a general factor. CFA parameters were estimated using a robust maximum likelihood estimation method. Models comparison was guided by the following criteria: (a) a qualitative evaluation of the fit indices of each model, (b) the CFI criterion, according to which if the difference in the CFIs between two nested models is smaller than |0.01|, the hypothesis of no difference in fit between the two competing models should not be rejected^37^ and (c) the scaled *χ*^2^ difference tests^38^. For the evaluation of model fit indices, two relative and two absolute fit indices, the Tucker Lewis index (TLI) and the comparative fit index (CFI), and the root mean square error of approximation (RMSEA) and the standardised root mean square residuals (SRMR), were computed. CFI and TLI values of >0.95 and >0.97, respectively, are considered as indicating acceptable and good fit. For RMSEA, values ranging from 0.05 to 0.08 reflect adequate fit; for SRMR, values <0.08 are considered a good fit^39^. We also computed RMSEA of the null model. If lower than 0.158, CFI and TLI may not be reliable and only RMSEA and SRMR were considered for evaluating the model fit^40^. The best-fitting factor structure was then tested in the UK-based sample.

#### Latent class analyses and cut-off scores

In order to test for the existence of distinct sensitivity categories, we performed a series of latent class analyses (LCAs) on all HSP items, testing models with 1–6 classes in subsamples A and B, as well as in the total primary sample. The optimal number of classes was determined based on the following criteria: (a) Akaike’s information criterion (AIC), (b) Bayesian information criterion (BIC), (c) Lo–Mendell–Rubin-adjusted likelihood ratio test (LMR-A) and^4^ Entropy. AIC and BIC are comparative indices, the lower the values the better the model. The LMR-A compares the fit of the specified class solution to a model with one fewer class. A significant *p*-value suggests that the specified model provides a better fit to the data than the more parsimonious model. Entropy refers to the confidence with which individuals can be categorised into the different classes, with values approaching 1 indicative of a clear delineation of membership^41^. After having identified the optimal number of classes, we investigated the distribution and overlap between the different sensitivity classes to suggest preliminary cut-off scores. Cut-off scores were identified based on the LCA results in subsample A (discovery sample) and applied to subsample B (replication sample) to test for specificity and sensitivity (i.e. the proportion of true positive and true negative classifications). In more detail, specificity and sensitivity were calculated based on the agreement between the categorisation based on the application of the cut-off scores from subsample A to subsample B and the categorisation based on the results of the LCA run on subsample B.

#### Characterisation of detected sensitivity groups

In order to investigate differences between the detected sensitivity groups, we first considered the bivariate associations in the follow-up sample, based on continuous scores, between sensitivity, the five personality traits and both negative and positive emotional reactivity. Significant associations were then followed up with multivariate analysis of variance (MANOVA) after grouping participants of the follow-up sample into low-sensitivity, medium-sensitivity and high-sensitivity groups based on the distribution of the groups that emerged in the primary sample. Statistically significant findings were further explored with Tukey post hoc tests.

All analyses were performed with the statistical software R (version 3.3.1) except for latent class analysis, which was run with Mplus (version 7.11). All data sets are available upon request from the corresponding author.

## Results

### Preliminary analysis

The distribution (i.e. estimated density) of the HSP mean score in the primary sample is available in the supplementary material section. Data appeared normally distributed with some indication for a bimodal pattern, given the observation of two emerging peaks with a small dip between them. Results of the confirmatory factor analyses for subsample A (*n* = 451) were the following: *χ*^2^ (324) = 1570, CFI = 679, TLI = 0.652, RMSEA = 0.085 [0.080–0.089], SRMR = 0.079 for the one-factor model; *χ*^2^ (321) = 1111, CFI = 798, TLI = 0.779, RMSEA = 0.068 [0.063 –0.072], SRMR = 0.080 for the three-factor model (for the three-factor solution, we maintained all 27 HSP items, including items 1 and 11, which were excluded in the analysis by Smolewska et al.^32^. The results remained statistically significant and unchanged when analyses were repeated without items 1 and 11), and *χ*^2^ (297) = 832, CFI = 860, TLI = 0.835, RMSEA = 0.058 [0.053–0.063], SRMR = 0.054 for the bifactor model. In subsample B (*n* = 450), fit indices were the following: *χ*^2^ (324) = 1571, CFI = 678, TLI = 0.651, RMSEA = 0.086 [0.081 –0.090], SRMR = 0.075 for the one-factor model; *χ*^2^ (321) = 1191, CFI = 0.775, TLI = 0.754, RMSEA = 0.072 [0.067–0.077], SRMR = 0.078 for the three-factor model and *χ*^2^ (297) = 870, CFI = 850, TLI = 0.823, RMSEA = 0.061 [0.056–0.066], SRMR = 0.052 for the bifactor model. Even though fit indices for the bifactor solution fell short on CFI and TLI in both subsamples, the solution was acceptable according to SRMR (lower than 0.07 at the upper bound) and RMSEA (lower than 0.08 at the upper bound), which are considered more reliable than CFI and TLI incremental indices for evaluating the model fit given that the RMSEA of the null model was 0.156 and 0.158 in subsample A and subsample B, respectively^40^. Furthermore, the bifactor solution was also significantly better than the one-factor and three-factor solution according to *∆*CFI (always >0.01) and to the scaled-*χ*^2^ difference (subsample A: *χ*^2^ DIFF(27) = 470, *p* < 0.001 for the comparison between bifactor vs. one-factor and *χ*^2^ DIFF(24) = 190, *p* < 0.001 for the comparison between bifactor vs. three-factor solution; subsample B: *χ*^2^DIFF(27) = 479, *p* < 0.001 for the comparison between bifactor vs. one-factor and *χ*^2^ DIFF(24) = 236, *p* < 0.001 for the comparison between bifactor vs. three-factor solution). These results suggest that the HSP scale reflects both three orthogonal (i.e. independent) scales and a general sensitivity factor across all items.

### Latent class analysis

Latent class analysis applied to subsample A supported a three-class solution which yielded a significant LMR-A at *p* < 0.04, adequate entropy (0.87) and lower BIC and AIC compared to the two-class model. The models with four to six classes had to be rejected, because none of them was significantly better than the three-class model. According to the three-class model, 31.27% of participants belong to a low-sensitive group, 42.15% to a medium-sensitive group and 26.58% to a highly sensitive group. Applied to subsample B, the three-class model (25.33% low sensitive, 44.67% medium sensitive and 30% highly sensitive) showed the same entropy value but although BIC and AIC values decreased from the two-class model to the three-class model, the LMR-A was only marginally significant. Finally, latent class analysis applied to the whole primary sample, confirming that the three-class model fits the data best (Table 1). Descriptive statistics for the HSP summary score and for EOE, AES and LST factors in the primary sample are provided in Table 2. Hence, our analyses suggest that there exist three rather than two sensitivity groups in the general population with a frequency distribution of approximately 30% in the low-sensitivity, 40% in the medium-sensitivity and 30% in the high-sensitivity group.

**Table 1:** Latent class analysis (primary sample)

Classes	AIC	BIC	BIC adj.	LMR-A (*p*)	Entropy
*Subsample A*					
One	46442.674	46664.674	46493.318		
Two	44450.773	44787.913	44527.676	2036.003 (<0.01)	0.92
**Three**	**44111.779**	**44564.040**	**44214.779**	**3927.700 (0.04)**	**0.87**
Four	43880.927	44448.310	44010.349	285.185 (0.39)	0.88
Five	43719.090	44401.594	43874.771	385.595 (0.11)	0.89
Six	43651.077	44448.701	43833.017	123.293 (0.69)	0.87
*Subsample B*					
One	46289.131	46520.131	46348.655		
Two	44434.179	4471.137	44510.901	1908.793 (<0.01)	0.89
**Three**	**43995.041**	**44447.059**	**44097.961**	**492.260 (0.08)**	**0.87**
Four	43821.822	44388.898	43950.938	227.888 (0.42)	0.87
Five	43635.428	44317.563	43790.742	3288.694 (0.77)	0.87
Six	43539.251	44336.445	43720.763	151.292 (0.27)	0.87
*Total sample*					
One	92730.870	92990.439	92818.943		
Two	88794.156	89188.043	88927.624	3919.613 (<0.01)	0.90
**Three**	**88009.905**	**88538.291**	**88188.949**	**835.862 (<0.01)**	**0.86**
Four	87672.875	87897.494	87897.494	390.978 (0.08)	0.87
Five	87341.458	88138.840	87611.651	387.873 (0.12)	0.85
Six	87070.632	88002.512	87386.400	325.110 (0.61)	0.85

The best-fitting solution is highlighted in bold

*AIC* Akaike’s information criterion, *BIC* Bayesian information criterion, *LMR-A* Lo–Mendell–Rubin-adjusted likelihood ratio test

**Table 2:** Sensitivity groups (primary sample)

	Frequencies	M (SD)			
		HSP	EOE	AES	LST
*Subsample A*					
Low sensitive	31.27%	3.17 (0.46)	3.27 (0.59)	2.02 (0.70)	4.13 (1.02)
Medium sensitive	42.15%	4.23 (0.35)	4.49 (0.47)	3.32 (0.72)	4.68 (0.81)
High sensitive	26.58%	5.10 (0.39)	5.32 (0.53)	4.68 (0.64)	5.09 (0.68)
*Subsample B*					
Low sensitive	25.33%	3.05 (0.45)	3.18 (0.57)	2.00 (0.59)	3.86 (0.83)
Medium sensitive	44.67%	4.04 (0.35)	4.32 (0.56)	2.90 (0.70)	4.64 (0.77)
High sensitive	30.00%	4.99 (0.45)	5.18 (0.67)	4.51 (0.73)	5.12 (0.72)
*Total sample*					
Low sensitive	30.52%	3.14 (0.45)	3.26 (0.57)	2.04 (0.64)	4.03 (0.91)
Medium sensitive	40.29%	4.14 (0.33)	4.43 (0.52)	3.09 (0.73)	4.67 (0.80)
High sensitive	29.19%	5.02 (0.43)	5.23 (0.60)	4.57 (0.68)	5.10 (0.71)

*HSP* highly sensitive Person scale (total score), *EOE* ease of excitation subscale, *AES* aesthetic sensitivity subscale, *LST* low sensory threshold subscale

### Cut-off scores

**Fig. 1:**
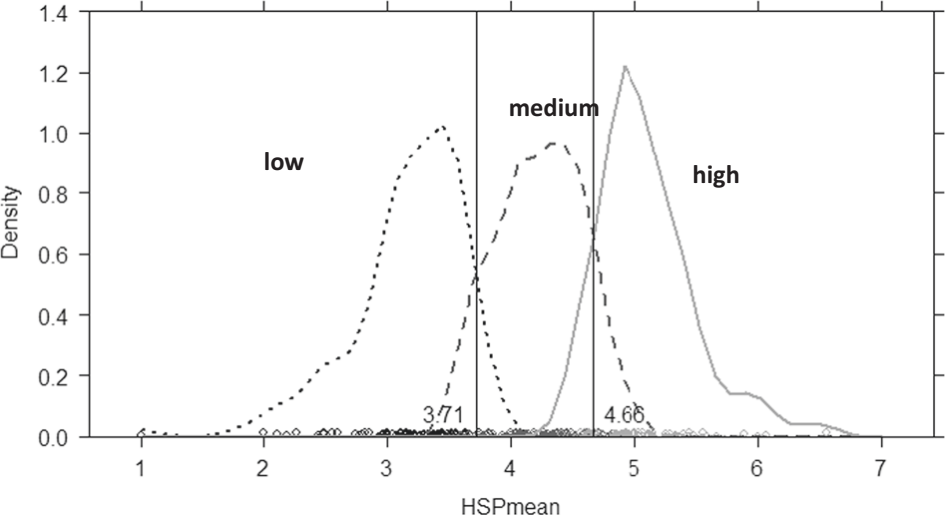
Distribution of the three sensitivity groups and associated cut-off scores based on the HSP total score in subsample A

In order to estimate cut-off scores, we identified the intersection points between the estimated densities of the low-sensitivity and medium-sensitivity (score = 3.71) and between the medium-sensitivity and high-sensitivity groups (score = 4.66; Fig. 1) in subsample A. These cut-off scores were then applied to subsample B using the LCA classification of subsample B as the reference criterion. Sensitivity and specificity values for the classification between high-sensitive and medium-sensitive individuals, with 0.93 and 0.82, respectively, and for medium-sensitive vs. low-sensitive ones with 0.74 and 0.96, respectively, were satisfactory. The factor structure of the HSP scale and existence of three sensitivity groups were replicated in a smaller independent sample of Stony Brook University undergraduate students (*N* = 417). Consistent with results of the main sample, the confirmatory factor analysis supported the bifactor solution as significantly better than the one-factor and three-factor solutions and the latent class analysis confirmed the existence of the same three sensitivity groups. In a further independent sample (*N* = 503) which featured a 5-point Likert scale instead of the 7-point scale, results supported the bifactor solution, but the latent class analysis results were less clear compared to the other samples (i.e. LMR-A test was significant for the two-class but not for the three-class solution). However, the three-class solution was almost identical to the sample of the main analysis.

### Characterisation of sensitivity groups

#### Personality

Bivariate correlations revealed a significant negative association between the HSP mean score (as well as subscales EOE and LST) and the personality trait of extraversion. There was also a significant positive association between HSP (and all subscales) and neuroticism (Table 3). In order to investigate differences in extraversion and neuroticism between the three sensitivity groups, we divided the follow-up sample into groups based on the distribution of the three sensitivity groups that emerged in the primary sample. Participants with HSP scores above the upper 30th percentile were categorised into the high-sensitivity group, those scoring below the lower 30th percentile into the low-sensitivity group and the remaining participants into the medium-sensitivity group (subsequent results were similar when creating groups by applying the cut-off scores reported earlier). According to MANOVA, controlling for age and gender, a significant group difference emerged (Pillai’s trace = 0.29, *F*(2,219) = 7.329, *p* < 0.001). Specifically, the three groups differed significantly regarding extraversion (*F*(2,227) = 6.82, *p* = 0.001) and neuroticism (*F*(2,227) = 44.94, *p* < 0.001). Tukey post hoc tests provided more insight into these differences, showing that extraversion was significantly lower in the high-sensitivity group compared to the low-sensitivity group (*p* < 0.001) and marginally significantly lower than the medium-sensitivity group (*p* = 0.07). For neuroticism, all three groups differed significantly from each other. The different group means for all personality traits are presented in Table 4.

**Table 3:** Bivariate associations (follow-up sample)

	1	2	3	4	5	6	7	8	9	10
1. HSP	—									
2. EOE	0.79**	—								
3. AES	0.57**	0.08	—							
4. LST	0.79**	0.46**	0.29**	—						
5. Extraversion	–0.24**	–.36**	0.11^#^	–0.19**	—					
6. Agreeableness	0.12	–0.03	0.28**	0.06	0.3**	—				
7. Conscientiousness	–0.01	–0.12	0.06	0.08	0.03	0.16*	—			
8. Neuroticism	0.56**	0.58**	0.15*	0.40**	–0.26**	0.05	–0.07	—		
9. Openness	0.01	–0.14*	0.37**	–0.14*	0.18**	0.04	0.06	–0.09	—	
10. Positive Reactivity	0.14*	0.19**	0.06	0.02	–0.11^#^	0.11^#^	–0.01	0.15*	–0.13*	—
11. Negative Reactivity	0.11^#^	0.12^#^	–0.01	0.12^#^	–0.02	0.11	0.14*	0.04	–0.08	0.24**

*HSP* highly sensitive Person scale (total score), *EOE* ease of excitation subscale, *AES* aesthetic sensitivity subscale, *LST* low sensory threshold subscale

*p < 0.05; **p < 0.01; ^#^*p* < 0.10

**Table 4:** Characterisation of sensitivity groups (follow-up sample)

	Sensitivity groups			ANOVA	
	Low *M* (SD)	Medium *M* (SD)	High *M* (SD)	*F*(2,227)	*p*
*Personality*					
Extraversion	3.23 (.86)	3.01 (.87)	2.73 (.81)	6.82**	<.01
Agreeableness	3.83 (.74)	3.91 (.46)	3.95 (.54)	0.77	0.46
Conscientiousness	3.42 (.63)	3.36 (.60)	3.41 (.66)	0.82	0.82
Neuroticism	2.61 (.66)	3.14 (.58)	3.60 (.62)	44.94**	<.01
Openness	3.68 (.56)	3.62 (.57)	3.72 (.54)	0.53	0.53
*Emotional reactivity*					
Positive reactivity	10.79 (18.23)	14.87 (20.84)	18.50 (22.01)	**2.42#**	0.09
Negative reactivity	30.12 (30.13)	36.14 (27.77)	36.37 (35.32)	0.93	0.40

The best-fitting solution is highlighted in bold

*p < 0.05; **p < 0.01; ^#^*p* < 0.10

#### Emotional reactivity

According to bivariate correlations, both the HSP mean score and the EOE subscale were positively associated with positive emotional reactivity. There was also a marginally significant positive association between HSP (and EOE subscale) and negative emotional reactivity. The difference in positive emotional reactivity between the three sensitivity groups approached significance (*F*(2,224) = 2.45, *p* = 0.09). However, even though group means appear to suggest a trend in the expected direction (i.e. the more sensitive the more reactive), groups did not differ significantly regarding negative emotional reactivity (see Table 4 for the different group means).

## Discussion

The current study set out to test for the existence of distinct sensitivity groups, as suggested by several theories on environmental sensitivity^7,14,15^ after clarifying whether the applied self-report measure—the HSP scale—is measuring a unitary concept of sensitivity. Furthermore, the study aimed at developing preliminary cut-off scores for the different detected sensitivity groups, as well as investigating differences between groups regarding common personality traits and emotional reactivity. This is one of the first empirical efforts to explore and confirm the existence of different sensitivity groups in an adult human sample (see also ref. ^42^), applying a modern data-driven statistical approach, based on the specific measurement of a general sensitivity trait rather than distal markers of sensitivity.

In a preliminary step, we investigated the structure of the HSP scale, given on-going disagreements over the last 10 years whether the scale reflects a unidimensional construct, as suggested by theory^14^ and first empirical data^14^, or multiple components of sensitivity as proposed by a large number of studies^32,43^. The confirmatory factor analysis supported a bifactor structure, which means that the HSP scale is made up of both a general sensitivity construct as well as three individual subscales which capture sensitivity to sensory stimuli (LST), sensitivity to overstimulation (EOE) and sensitivity to the aesthetic quality of the environment (AES). This result reconciles the two contradictory views suggesting that they are both simultaneously valid rather than mutually exclusive and also provides statistical justification for the use of the mean score across all items as a measure of general environmental sensitivity. As discussed elsewhere in more detail^5^, individual differences in general environmental sensitivity tend to manifest themselves both in response to contextual adversity (i.e. the dark side of susceptibility or the vulnerability component of the Diathesis-Stress model^11^) as well as in response to the beneficial effects of positive environmental factors (i.e. the bright side of susceptibility as conceptualised in the Vantage Sensitivity model^44^). In other words, more sensitive individuals tend to be more reactive to both adverse and supportive exposures, whereas less-sensitive individuals are less reactive to threat but also less likely to benefit from positive aspects of the environment. Both of these sensitivity aspects seem to be captured by the bifactor structure of the scale and are, consequently, reflected in the HSP total score.

The main contribution of this paper is the identification of distinct sensitivity groups. In contrast to current theories, the latent class analysis suggests that there are three rather than two sensitivity groups. Importantly, these results are in line with what has been recently reported in children and adolescents^17^. The existence of a high-sensitive group making up about 31% of the population is consistent with all theories on environmental sensitivity^6,7,14,15,18,20,45^, as well as a large number of empirical studies reporting that a minority of the population appears to be highly sensitive^7,14,21–25^. However, our findings suggest that the less-than-highly sensitive individuals fall into two distinct groups rather than just one. About 40% of the population are characterised by medium sensitivity, while ~29% make up a group that is particularly low in sensitivity. These results suggest that sensitivity is not a binary trait as implied by multiple theories^13,46^ and empirical research on reactivity/responsivity in various animal models^47,48^. Consequently, the dichotomous metaphor of 'dandelions' vs. 'orchids' —though intuitively comprehensible and helpful when explaining individual differences in environmental sensitivity—is not supported by this study. Although our analysis supports the existence of highly sensitive or responsive individuals (i.e. orchids), the story regarding 'dandelions' is more complicated because they can be further divided into two categories. If we consider 'dandelions' as the metaphorical example of the low-sensitive group, what plant species best reflects the medium-sensitive group? Sticking to the well-known flower metaphor, we suggest 'tulips' as a prototypical example for medium sensitivity. Tulips are very common, but less fragile than orchids while more sensitive to climate than dandelions. In summary, while some people are highly sensitive (i.e. orchids), the majority have a medium sensitivity (i.e. tulips) and a substantial minority are characterised by a particularly low sensitivity (i.e. dandelions).

Investigation of HSP total and subscale scores across the three different sensitivity groups suggests that they differ in degree of sensitivity rather than relative composition of HSP components, given that the means across all subscales were consistently the lowest in dandelions, intermediate in tulips and the highest in orchids. Hence, the three different groups seem to remain along a continuum of general sensitivity—which itself appears to be a quantitative and normally distributed trait. The finding that the relative composition of HSP components is similar across all groups also implies that the same or similar mechanisms may underlie sensitivity in each of the groups. In other words, the neurophysiological and psychological factors that drive environmental sensitivity^5,49^, are probably similar across groups, but more strongly pronounced and manifested in some people (i.e. orchids) and less in others (i.e. tulips followed by dandelions).

Although the group-specific mean scores of the HSP total and subscales suggest that differences between the three detected sensitivity groups may be more of quantitative than qualitative nature, we did find significant group differences in terms of common personality traits and emotional reactivity. According to these follow-up analyses, the three groups differed regarding personality traits extraversion and neuroticism, as well as emotional reactivity to positive mood induction. Orchids had significantly higher levels of neuroticism and positive emotional reactivity while scoring lower in extraversion than the other groups. Dandelions, on the other hand, had higher levels of extraversion but lower scores of neuroticism and positive emotional reactivity. Although group means also suggested a trend for negative emotional reactivity with orchids being most reactive to sad mood induction, followed by tulips and dandelions, differences did not reach statistical significance. In summary, these additional analyses suggest that orchids tend to be more introverted and prone to negative effect (neuroticism) than the other groups. However, they also appear to show a stronger emotional response to positive experiences. Dandelions are more extroverted and less anxious but at the same time less responsive to positive mood induction. Finally, tulips occupy the middle ground regarding all these traits.

One important implication of the detected sensitivity groups is that orchids may be more responsive to psychological intervention than to dandelions due to their heightened sensitivity to positive exposures (i.e. positive emotional reactivity). Recent studies on sensitivity in children and adolescents provide first evidence that this is indeed the case. For example, HSC was found to predict treatment response to a school-based resilience-promoting intervention^50^ with children scoring in the top 25% of the sensitivity scale (i.e. orchids) benefitting from the intervention regarding the reduction of depression symptoms, while those in the bottom 25% of the scale (i.e. dandelions) completely failed to do so^51^. Similar findings emerged in a study investigating whether sensitivity moderated the effects of a school-based anti-bullying intervention in a large randomised controlled trial involving 2042 children. Treatment effects on reduction of victimisation and internalising symptoms were significant in boys scoring in the top 25% of HSC (i.e. orchids), whereas boys in the bottom 25% of HSC (i.e. dandelions) did not show any improvement in response to the intervention^52^.

While the majority of research in the field of environmental sensitivity tends to target the more sensitive individuals^7,21^, future research should also focus on those individuals that make up the medium-sensitive and low-sensitive groups. While less-sensitive individuals (i.e. dandelions) may be more resilient in the face of adversity, they also appear to suffer the disadvantage of being more resistant to positive effects of intervention^51,52^. Hence, a better understanding of low sensitivity may be particularly important when investigating individual differences in treatment response.

Although the preliminary cut-off scores that we identified await replication in independent samples, they may allow researchers to further explore the specific features that characterise the detected sensitivity groups while also enabling practitioners to apply the HSP measure in order to assess sensitivity on an individual level. The a priori identification of more and less-sensitive individuals may represent a crucial step en route towards more personalised intervention programmes. Of course, we have to acknowledge that these cut-off scores are mere approximations, given less-than-perfect reliability of any self-report measure. Furthermore, cut-offs may very well differ between populations. However, even an approximate identification of low-sensitive, medium-sensitive and high-sensitive individuals may be of significant value, given recent findings that environmental sensitivity predicts individual differences in treatment response^51,52^.

The current paper has multiple strengths, including multiple samples, a cross-validation approach, application of hypothesis-free data-driven statistical procedures in order to identify different sensitivity groups and behavioural data for the assessment of emotional reactivity. However, our findings should be considered in light of the limitation that the data were based on one self-reported psychological indicator of environmental sensitivity. Future research should expand similar analyses to other more objective markers of environmental sensitivity (e.g. physiological reactivity, brain imaging, etc.) and in cross-cultural studies.

In conclusion, besides providing evidence that the HSP scale reflects indeed a unitary dimension of environmental sensitivity, we identified three sensitivity groups in the general population rather than the two proposed by common theories on individual differences in environmental sensitivity. In addition to high-sensitive (i.e. orchids) and low-sensitive (i.e. dandelions) individuals, we also detected a group representing individuals with medium sensitivity (i.e. tulips). Orchids are characterised by higher neuroticism and lower extraversion while being more susceptible to positive mood induction. Dandelions are more extraverted and score lower on neuroticism but also have a lower positive emotional reactivity with tulips being situated between dandelions and orchids.

## Electronic supplementary material

Density distribution of the HSP total score
