# Supplementary figures and images for: Dandelions, tulips and orchids: evidence for the existence of low-sensitive, medium-sensitive and high-sensitive individuals
Source: Transl Psychiatry. 2018 Jan 22;8:24. doi: 10.1038/s41398-017-0090-6 (PMC5802697; doi:10.1038/s41398-017-0090-6)

**Supplementary materials**

Density distribution of theHSP total score (*N* = 901)


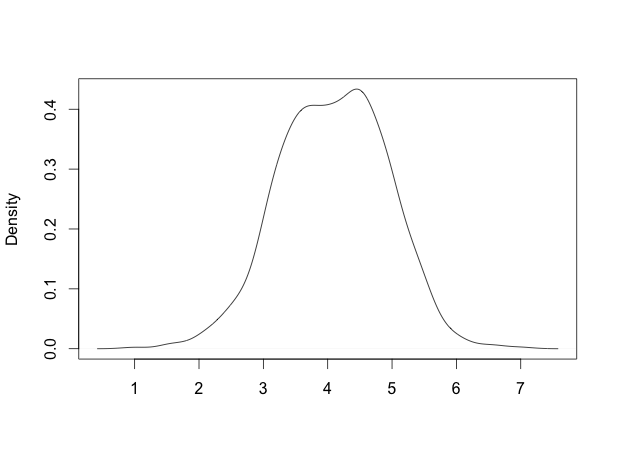

Supplement: Supplementary file 1 — Density distribution of the HSP total score [file 41398_2017_90_MOESM1_ESM.docx]
